# Benzyl 3-[(*E*)-benzyl­idene]dithio­carbazate

**DOI:** 10.1107/S1600536808012944

**Published:** 2008-05-07

**Authors:** Shang Shan, Yu-Liang Tian, Shan-Heng Wang, Wen-Long Wang, Ying-Li Xu

**Affiliations:** a College of Chemical Engineering and Materials Science, Zhejiang University of Technology, People’s Republic of China

## Abstract

Crystals of the title compound, C_15_H_14_N_2_S_2_, were obtained from a condensation reaction of benzyl dithio­carbazate and benzaldehyde. The mol­ecule assumes an *E* configuration about the N=C double bond. The phenyl ring of the thio­ester group is nearly perpendicular to the dithio­carbazate plane, with a dihedral angle of 84.60 (5)°. In the crystal structure, inter­molecular N—H⋯S hydrogen bonding links adjacent mol­ecules to form a centrosymmetric supra­molecular dimer.

## Related literature

For general background, see: Okabe *et al.* (1993 ▶); Hu *et al.* (2001 ▶). For related structures, see: Shan *et al.* (2006 ▶, 2008 ▶); Zhang *et al.* (2005 ▶). For synthesis, see: Hu *et al.* (2001 ▶).
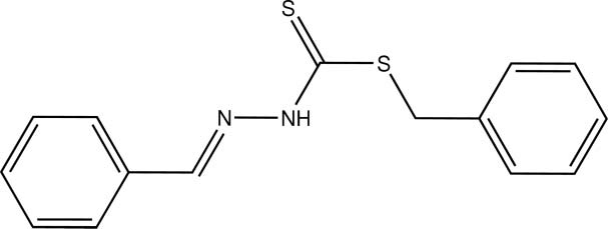


## Experimental

### 

#### Crystal data

C_15_H_14_N_2_S_2_
*M* *_r_* = 286.40
Monoclinic, 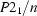

*a* = 5.0053 (18) Å
*b* = 23.075 (8) Å
*c* = 12.646 (5) Å
β = 97.652 (12)°
*V* = 1447.6 (9) Å^3^
*Z* = 4
Mo *K*α radiation
μ = 0.35 mm^−1^
*T* = 295 (2) K
0.30 × 0.28 × 0.22 mm

#### Data collection

Rigaku R-AXIS RAPID diffractometer
Absorption correction: none
15395 measured reflections
2653 independent reflections
2115 reflections with *I* > 2σ(*I*)
*R*_int_ = 0.034

#### Refinement

*R*[*F* ^2^ > 2σ(*F* ^2^)] = 0.032
*wR*(*F* ^2^) = 0.087
*S* = 1.05
2653 reflections
172 parameters
H-atom parameters constrained
Δρ_max_ = 0.15 e Å^−3^
Δρ_min_ = −0.18 e Å^−3^

### 

Data collection: *PROCESS-AUTO* (Rigaku, 1998 ▶); cell refinement: *PROCESS-AUTO*; data reduction: *CrystalStructure* (Rigaku/MSC, 2002 ▶); program(s) used to solve structure: *SIR92* (Altomare *et al.*, 1993 ▶); program(s) used to refine structure: *SHELXL97* (Sheldrick, 2008 ▶); molecular graphics: *ORTEP-3 for Windows* (Farrugia, 1997 ▶); software used to prepare material for publication: *WinGX* (Farrugia, 1999 ▶).

## Supplementary Material

Crystal structure: contains datablocks I, global. DOI: 10.1107/S1600536808012944/xu2422sup1.cif

Structure factors: contains datablocks I. DOI: 10.1107/S1600536808012944/xu2422Isup2.hkl

Additional supplementary materials:  crystallographic information; 3D view; checkCIF report

## Figures and Tables

**Table 1:** Hydrogen-bond geometry (Å, °)

*D*—H⋯*A*	*D*—H	H⋯*A*	*D*⋯*A*	*D*—H⋯*A*
N2—H2*N*⋯S1^i^	0.86	2.56	3.396 (2)	165

Symmetry code: (i) 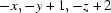.

**Fig. 1.**
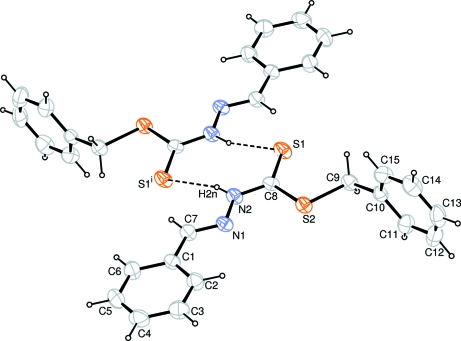
The molecular structure of the title compound with 30% probability displacement ellipsoids (arbitrary spheres for H atoms). Dashed lines indicate hydrogen bonding [symmetry code: (i) -x,1 - y,2 - z].

## supplementary crystallographic information

### Comment

Hydrazone and its derivatives have attracted our much attention as they showed the potential application in biological field (Okabe *et al.*, 1993; Hu *et al.*, 2001). As part of ongoing investigation on anti-cancer compounds the title compound has been prepared and its crystal structure is presented here.

The molecular structure of the title compound is shown in Fig. 1. The N1—C7 bond distance (Table 1) indicates a typical C═N double bond; around the C═N bond the molecule assumes an E-configuration, similar to that found in methyl (β-*N*-phenylmethylene)dithiocarbazate (Shan *et al.*, 2006). The dithiocarbazate moiety is coplanar with the C1-phenyl ring, the dihedral angle of 0.99 (11)° agrees with 3.00 (6)° found in methyl β-*N*-nitrophenylmethylenedithiocarbazate (Shan *et al.*, 2008). In the thioester group, the C10-phenyl ring is nearly perpendicular to the dithiocarbazate plane with a dihedral angle of 84.60 (5)°. The S2—C8—N2 bond angle of 113.71 (12)° is much smaller than the S1—C8—N2 bond angle of 121.27 (13)°, which agrees with those found in related structures (Shan *et al.*, 2006; Zhang *et al.*, 2005).

In the crystal structure, adjacent molecules are linked into a centro-symmetric supra-molecular dimer by intermolecular N—H···S hydrogen bonding (Fig. 1 and Table 2).

### Experimental

Benzyl dithiocarbazate was synthesized in the manner reported previously (Hu *et al.*, 2001). Benzyl dithiocarbazate (1.98 g, 10 mmol) and benzaldehyde (1.06 g, 10 mmol) were dissolved in ethanol (40 ml) and the solution was refluxed for 12 h. Yellow crystalline product appeared after cooling to room temperature. They were separated and washed with cold water three times. Single crystals of the title compound were obtained by recrystallization from an ethanol solution.

### Refinement

H atoms were placed in calculated positions with C—H = 0.97 (methylene), 0.93 Å (aromatic) and N—H = 0.86 Å, and refined in riding mode with *U*_iso_(H) = 1.2*U*_eq_(C,N)

### Crystal data

**Table:**

C_15_H_14_N_2_S_2_	*F*_000_ = 600
*M**_r_* = 286.40	*D*_x_ = 1.314 Mg m^−^^3^
Monoclinic, *P*2_1_/*n*	Mo *K*α radiation λ = 0.71073 Å
Hall symbol: -P 2yn	Cell parameters from 6568 reflections
*a* = 5.0053 (18) Å	θ = 1.9–25.0º
*b* = 23.075 (8) Å	µ = 0.36 mm^−^^1^
*c* = 12.646 (5) Å	*T* = 295 (2) K
β = 97.652 (12)º	Prism, yellow
*V* = 1447.6 (9) Å^3^	0.30 × 0.28 × 0.22 mm
*Z* = 4	

### Data collection

**Table:**

Rigaku R-AXIS RAPID diffractometer	2653 independent reflections
Radiation source: fine-focus sealed tube	2115 reflections with *I* > 2σ(*I*)
Monochromator: graphite	*R*_int_ = 0.034
Detector resolution: 10.00 pixels mm^-1^	θ_max_ = 25.5º
*T* = 295(2) K	θ_min_ = 1.8º
ω scans	*h* = −5→6
Absorption correction: none	*k* = −27→27
15395 measured reflections	*l* = −15→13

### Refinement

**Table:**

Refinement on *F*^2^	Secondary atom site location: difference Fourier map
Least-squares matrix: full	Hydrogen site location: inferred from neighbouring sites
*R*[*F*^2^ > 2σ(*F*^2^)] = 0.032	H-atom parameters constrained
*wR*(*F*^2^) = 0.087	*w* = 1/[σ^2^(*F*_o_^2^) + (0.0424*P*)^2^ + 0.226*P*] where *P* = (*F*_o_^2^ + 2*F*_c_^2^)/3
*S* = 1.05	(Δ/σ)_max_ = 0.001
2653 reflections	Δρ_max_ = 0.15 e Å^−^^3^
172 parameters	Δρ_min_ = −0.18 e Å^−^^3^
Primary atom site location: structure-invariant direct methods	Extinction correction: none

### Special details

**Table:**

Geometry. All e.s.d.'s (except the e.s.d. in the dihedral angle between two l.s. planes) are estimated using the full covariance matrix. The cell e.s.d.'s are taken into account individually in the estimation of e.s.d.'s in distances, angles and torsion angles; correlations between e.s.d.'s in cell parameters are only used when they are defined by crystal symmetry. An approximate (isotropic) treatment of cell e.s.d.'s is used for estimating e.s.d.'s involving l.s. planes.
Refinement. Refinement of *F*^2^ against ALL reflections. The weighted *R*-factor *wR* and goodness of fit *S* are based on *F*^2^, conventional *R*-factors *R* are based on *F*, with *F* set to zero for negative *F*^2^. The threshold expression of *F*^2^ > σ(*F*^2^) is used only for calculating *R*-factors(gt) *etc*. and is not relevant to the choice of reflections for refinement. *R*-factors based on *F*^2^ are statistically about twice as large as those based on *F*, and *R*- factors based on ALL data will be even larger.

### Fractional atomic coordinates and isotropic or equivalent isotropic displacement parameters (Å^2^)

**Table:**

	*x*	*y*	*z*	*U*_iso_*/*U*_eq_	
S1	−0.16273 (11)	0.58470 (2)	0.94815 (4)	0.06114 (17)	
S2	0.02011 (10)	0.602875 (18)	0.73176 (4)	0.05159 (15)	
N1	0.3232 (3)	0.50304 (6)	0.78197 (11)	0.0493 (4)	
N2	0.1740 (3)	0.51871 (6)	0.86146 (11)	0.0516 (4)	
H2N	0.1829	0.4979	0.9183	0.062*	
C1	0.6444 (3)	0.43585 (7)	0.72802 (14)	0.0486 (4)	
C2	0.6602 (4)	0.46157 (8)	0.62992 (16)	0.0599 (5)	
H2	0.5621	0.4951	0.6109	0.072*	
C3	0.8199 (5)	0.43796 (10)	0.56054 (17)	0.0721 (6)	
H3	0.8285	0.4554	0.4948	0.087*	
C4	0.9663 (4)	0.38875 (10)	0.5882 (2)	0.0764 (6)	
H4	1.0736	0.3728	0.5411	0.092*	
C5	0.9551 (4)	0.36326 (10)	0.68450 (19)	0.0766 (6)	
H5	1.0555	0.3300	0.7029	0.092*	
C6	0.7955 (4)	0.38637 (8)	0.75514 (17)	0.0632 (5)	
H6	0.7894	0.3687	0.8209	0.076*	
C7	0.4749 (4)	0.45888 (7)	0.80289 (15)	0.0529 (4)	
H7	0.4769	0.4407	0.8687	0.064*	
C8	0.0160 (3)	0.56550 (7)	0.85190 (13)	0.0458 (4)	
C9	−0.2180 (4)	0.66100 (7)	0.74434 (14)	0.0531 (4)	
H9A	−0.3989	0.6455	0.7419	0.064*	
H9B	−0.1716	0.6810	0.8117	0.064*	
C10	−0.2041 (4)	0.70207 (7)	0.65275 (14)	0.0494 (4)	
C11	−0.3930 (4)	0.69963 (9)	0.56287 (17)	0.0667 (5)	
H11	−0.5313	0.6725	0.5594	0.080*	
C12	−0.3804 (5)	0.73660 (11)	0.47831 (19)	0.0829 (7)	
H12	−0.5085	0.7340	0.4181	0.099*	
C13	−0.1794 (5)	0.77726 (10)	0.4826 (2)	0.0791 (7)	
H13	−0.1727	0.8028	0.4261	0.095*	
C14	0.0112 (5)	0.77991 (9)	0.5708 (2)	0.0763 (6)	
H14	0.1498	0.8069	0.5738	0.092*	
C15	−0.0015 (4)	0.74263 (8)	0.65529 (17)	0.0646 (5)	
H15	0.1288	0.7449	0.7149	0.077*	

### Atomic displacement parameters (Å^2^)

**Table:**

	*U*^11^	*U*^22^	*U*^33^	*U*^12^	*U*^13^	*U*^23^
S1	0.0859 (4)	0.0527 (3)	0.0517 (3)	0.0075 (2)	0.0347 (3)	0.0005 (2)
S2	0.0620 (3)	0.0500 (3)	0.0471 (3)	0.00360 (19)	0.0232 (2)	0.00234 (19)
N1	0.0549 (9)	0.0477 (8)	0.0489 (8)	−0.0014 (7)	0.0201 (7)	−0.0032 (6)
N2	0.0653 (9)	0.0477 (8)	0.0461 (8)	0.0036 (7)	0.0230 (7)	0.0024 (6)
C1	0.0480 (10)	0.0471 (9)	0.0524 (11)	−0.0032 (7)	0.0134 (8)	−0.0040 (8)
C2	0.0651 (12)	0.0590 (10)	0.0583 (12)	0.0062 (9)	0.0185 (10)	0.0013 (9)
C3	0.0781 (15)	0.0856 (15)	0.0573 (13)	0.0032 (12)	0.0262 (11)	−0.0024 (11)
C4	0.0655 (14)	0.0940 (16)	0.0730 (16)	0.0107 (12)	0.0213 (11)	−0.0231 (13)
C5	0.0759 (15)	0.0712 (13)	0.0842 (17)	0.0250 (11)	0.0162 (12)	−0.0062 (12)
C6	0.0666 (13)	0.0602 (11)	0.0645 (13)	0.0083 (9)	0.0144 (10)	0.0046 (9)
C7	0.0610 (11)	0.0505 (9)	0.0502 (11)	0.0008 (8)	0.0179 (9)	0.0027 (8)
C8	0.0546 (10)	0.0411 (8)	0.0442 (10)	−0.0077 (7)	0.0156 (8)	−0.0042 (7)
C9	0.0545 (11)	0.0533 (10)	0.0550 (11)	0.0019 (8)	0.0197 (9)	−0.0007 (8)
C10	0.0528 (10)	0.0460 (9)	0.0519 (11)	0.0087 (8)	0.0166 (8)	−0.0021 (8)
C11	0.0584 (12)	0.0711 (13)	0.0704 (14)	0.0066 (10)	0.0081 (10)	0.0072 (11)
C12	0.0804 (16)	0.0956 (17)	0.0710 (15)	0.0220 (14)	0.0040 (12)	0.0202 (13)
C13	0.0980 (18)	0.0725 (14)	0.0732 (16)	0.0309 (13)	0.0347 (14)	0.0260 (12)
C14	0.0918 (17)	0.0560 (11)	0.0876 (17)	−0.0046 (11)	0.0362 (14)	0.0068 (11)
C15	0.0734 (13)	0.0599 (11)	0.0619 (13)	−0.0063 (10)	0.0146 (10)	−0.0004 (9)

### Geometric parameters (Å, °)

**Table:**

S1—C8	1.6636 (17)	C5—H5	0.9300
S2—C8	1.7495 (17)	C6—H6	0.9300
S2—C9	1.8153 (18)	C7—H7	0.9300
N1—C7	1.277 (2)	C9—C10	1.505 (2)
N1—N2	1.3777 (19)	C9—H9A	0.9700
N2—C8	1.334 (2)	C9—H9B	0.9700
N2—H2N	0.8600	C10—C15	1.377 (3)
C1—C2	1.387 (3)	C10—C11	1.379 (3)
C1—C6	1.387 (3)	C11—C12	1.376 (3)
C1—C7	1.454 (2)	C11—H11	0.9300
C2—C3	1.375 (3)	C12—C13	1.371 (3)
C2—H2	0.9300	C12—H12	0.9300
C3—C4	1.372 (3)	C13—C14	1.370 (3)
C3—H3	0.9300	C13—H13	0.9300
C4—C5	1.360 (3)	C14—C15	1.380 (3)
C4—H4	0.9300	C14—H14	0.9300
C5—C6	1.382 (3)	C15—H15	0.9300
			
C8—S2—C9	101.79 (8)	N2—C8—S2	113.71 (12)
C7—N1—N2	115.03 (14)	S1—C8—S2	125.02 (10)
C8—N2—N1	121.23 (14)	C10—C9—S2	107.43 (11)
C8—N2—H2N	119.4	C10—C9—H9A	110.2
N1—N2—H2N	119.4	S2—C9—H9A	110.2
C2—C1—C6	118.64 (17)	C10—C9—H9B	110.2
C2—C1—C7	122.28 (16)	S2—C9—H9B	110.2
C6—C1—C7	119.08 (17)	H9A—C9—H9B	108.5
C3—C2—C1	120.55 (19)	C15—C10—C11	117.96 (18)
C3—C2—H2	119.7	C15—C10—C9	121.28 (17)
C1—C2—H2	119.7	C11—C10—C9	120.75 (17)
C4—C3—C2	120.1 (2)	C12—C11—C10	121.2 (2)
C4—C3—H3	120.0	C12—C11—H11	119.4
C2—C3—H3	120.0	C10—C11—H11	119.4
C5—C4—C3	120.2 (2)	C13—C12—C11	120.2 (2)
C5—C4—H4	119.9	C13—C12—H12	119.9
C3—C4—H4	119.9	C11—C12—H12	119.9
C4—C5—C6	120.5 (2)	C14—C13—C12	119.4 (2)
C4—C5—H5	119.7	C14—C13—H13	120.3
C6—C5—H5	119.7	C12—C13—H13	120.3
C5—C6—C1	120.0 (2)	C13—C14—C15	120.3 (2)
C5—C6—H6	120.0	C13—C14—H14	119.9
C1—C6—H6	120.0	C15—C14—H14	119.9
N1—C7—C1	122.63 (16)	C10—C15—C14	121.0 (2)
N1—C7—H7	118.7	C10—C15—H15	119.5
C1—C7—H7	118.7	C14—C15—H15	119.5
N2—C8—S1	121.27 (13)		

### Hydrogen-bond geometry (Å, °)

**Table:**

*D*—H···*A*	*D*—H	H···*A*	*D*···*A*	*D*—H···*A*
N2—H2N···S1^i^	0.86	2.56	3.396 (2)	165

Symmetry codes: (i) −*x*, −*y*+1, −*z*+2.
